# Distinct Incidence of Takotsubo Syndrome Between Amyotrophic Lateral Sclerosis and Synucleinopathies: A Cohort Study

**DOI:** 10.3389/fneur.2018.01099

**Published:** 2018-12-13

**Authors:** Yuishin Izumi, Ryosuke Miyamoto, Koji Fujita, Yuki Yamamoto, Hirotsugu Yamada, Tomoyasu Matsubara, Yuki Unai, Ai Tsukamoto, Naoko Takamatsu, Hiroyuki Nodera, Shinya Hayashi, Masaya Oda, Atsuko Mori, Yoshihiko Nishida, Shunsuke Watanabe, Hirohisa Ogawa, Hisanori Uehara, Shigeo Murayama, Masataka Sata, Ryuji Kaji

**Affiliations:** ^1^Department of Clinical Neuroscience, Institute of Biomedical Sciences, Tokushima University Graduate School, Tokushima, Japan; ^2^Department of Community Medicine for Cardiology, Tokushima University Graduate School of Biomedical Sciences, Tokushima, Japan; ^3^Department of Neuropathology, Tokyo Metropolitan Geriatric Hospital and Institute of Gerontology, Tokyo, Japan; ^4^Department of Neurology, Tokushima Hospital, Yoshinogawa, Japan; ^5^Department of Internal Medicine, Kaminaka Hospital, Naka, Japan; ^6^Department of Neurology, Mifukai Vihara Hananosato Hospital, Miyoshi, Japan; ^7^Department of Neurology, Itsuki Hospital, Tokushima, Japan; ^8^Department of Pathology and Laboratory Medicine, Institute of Biomedical Sciences, Tokushima University Graduate School, Tokushima, Japan; ^9^Division of Pathology, Tokushima University Hospital, Tokushima, Japan; ^10^Department of Cardiovascular Medicine, Institute of Biomedical Sciences, Tokushima University Graduate School, Tokushima, Japan

**Keywords:** takotsubo syndrome, amyotrophic lateral sclerosis, synucleinopathy, Parkinson's disease, dementia with Lewy bodies, multiple system atrophy, sympathetic nerve, noradrenaline

## Abstract

Takotsubo syndrome (TTS) is an acute cardiac syndrome characterized by regional left ventricular dysfunction with a peculiar circumferential pattern, which typically results in apical ballooning. Evidence indicates a pivotal role of catecholamines in TTS, and researchers have discussed multiple hypotheses on the etiology, including multivessel coronary spasm, myocardial stunning, excessive transient ventricular afterload, and cardiac sympathetic overactivity with local noradrenaline spillover. Although central nervous system disorders, such as stroke and epilepsy, are known to trigger TTS, the incidence and clinical features of TTS in neurodegenerative disorders are poorly understood. Here, we retrospectively examined TTS cases in a single-center cohort composed of 250 patients with amyotrophic lateral sclerosis (ALS) and 870 patients with synucleinopathies [582 patients with Parkinson's disease (PD), 125 patients with dementia with Lewy bodies (DLB), and 163 patients with multiple system atrophy (MSA)] and identified 4 (1.6%, including 2 women) cases with ALS and no cases with synucleinopathies. Two ALS patients underwent autopsy and the pathological findings were compatible with the chronological changes identified in catecholamine-induced cardiomyopathy. A literature review identified 16 TTS cases with ALS, 1 case each with PD and DLB, and no cases with MSA. When current and previous TTS cases with ALS were concatenated: 55% (11/20) were female; 35% (7/20) had a bulbar-onset and 45% (9/20) had a limb-onset; the mean age of TTS onset was 63.3 ± 9.0 years and the mean interval time from ALS onset to TTS development was 4.9 ± 3.0 years; no (0/16) patients developed TTS within 12 months after ALS onset; 50% (10/20) underwent artificial ventilations; the mortality was 17% (3/18); and most cases had precipitating factors, and TTS development was associated with gastrostomy, tracheostomy, or infections in 45% (9/20) of the patients. This study demonstrated that ALS is a considerable predisposing factor of TTS and that synucleinopathies rarely cause TTS. The distinct TTS incidence between ALS and synucleinopathies may be due to cardiac sympathetic overactivity in ALS and may also be affected by cardiac sympathetic denervation in synucleinopathies. Moreover, the etiology of TTS in ALS may be reasonably explained by the two-hit theory.

## Introduction

Takotsubo syndrome (TTS) is an acute cardiac syndrome characterized by regional left ventricular dysfunction with a peculiar circumferential pattern, which typically results in apical ballooning of the left ventricle at end systole. The abnormality extends beyond the coronary artery supply regions, which distinguishes TTS from acute coronary syndrome. A wide variety of emotional and physical stress, including anger, shock, and illness, triggers TTS, and female sex is a strong predisposing factor for TTS development (1, 2).

Multiple lines of evidence have demonstrated the pivotal role of catecholamines in developing TTS (3–8), and researchers have proposed and discussed various hypotheses on the etiology, including multivessel coronary spasm, myocardial stunning, and excessive transient ventricular afterload (1). A recent review also suggested that cardiac sympathetic overactivity and local noradrenaline spillover may be fundamental in the etiology (9).

Central nervous system (CNS) disorders may cause TTS, and stroke and epilepsy are the most frequent causes among these disorders. In a review of TTS in CNS disorders, most TTS cases developed with acute-onset or paroxysmal CNS disorders (3). A unique exception is amyotrophic lateral sclerosis (ALS), which is a neurodegenerative disorder characterized by chronic, progressive upper and lower motor neuron death. The intriguing link between ALS and TTS may be explained by the increased cardiac noradrenergic activities that ALS patients continuously endure (10, 11). In contrast, synucleinopathies, including Parkinson's disease (PD), dementia with Lewy bodies (DLB), and multiple system atrophy (MSA), have rarely been reported to cause TTS (3), although they are also physically and mentally disabling and substantially more prevalent neurodegenerative disorders. The precise reason is unknown; however, it may be due to underreporting and no cohort study has investigated the TTS incidence in neurodegenerative disorders, with the exception of ALS.

The aim of this study is to retrospectively identify patients complicated by TTS in our cohort of ALS and synucleinopathies and assess the difference in the TTS incidence between these two entities. We also performed a literature review that focused on TTS cases accompanied by ALS or synucleinopathies.

## Materials and Methods

### Subjects

This study was approved by the Ethics Committee of the Tokushima University Hospital and written informed consent was obtained from the participant for the publication of this case report. We identified cases using medical records at the Tokushima University Hospital between June 2006 and December 2017, which included 250 patients with ALS and 870 patients with synucleinopathies (PD, 582; DLB, 125; MSA, 163). The latest patient accrual was September 2016. The diagnosis of ALS, PD, DLB, and MSA was made based on the revised El Escorial criteria (12), UK Brain Bank criteria (13), third report of the DLB Consortium (14), and **second** consensus statement on the diagnosis of MSA (15), respectively. TTS was diagnosed on the basis of the International Takotsubo Diagnostic Criteria (2).

### Statistical Analysis

Differences in the incidence of TTS between ALS and synucleinopathies were tested for significance using Fisher's exact test and the Haldane–Anscombe correction was used for calculating the odds ratio. The test was 2-sided and the limit for statistical significance (*P*) was set at 0.05. Statistical analysis was performed using R version 3.5.1. (R Foundation for Statistical Computing, Vienna, Austria) Data is presented as mean ± standard deviation, unless stated otherwise.

### Literature Review

A review of the literature was conducted to identify published cases of TTS with ALS or synucleinopathies (PD, DLB, and MSA). We searched PubMed and Google Scholar to identify relevant articles using the following MeSH terms: ALS, motor neuron disease, Parkinson disease, Parkinson's disease, Lewy body disease, dementia with Lewy bodies, multiple system atrophy, olivopontocerebellar atrophy, striatonigral degeneration, Shy-Drager syndrome, takotsubo cardiomyopathy, tako-tsubo cardiomyopathy, takotsubo syndrome, and tako-tsubo syndrome. We also manually examined the reference lists in relevant articles.

## Results

Four (1.6%, 4 of 250) patients with ALS developed TTS during the evaluation period, whereas no patients with synucleinopathies developed TTS. The incidence of TTS was significantly higher in the patients with ALS than in those with synucleinopathies (odds ratio 31.78, and 95% confidence interval 1.71–592.38, *P* = 0.002). Of the 4 ALS patients with TTS, 2 patients were women, and the mean age at TTS onset was 65.8 ± 9.6 (range, 55–78) years. The mean interval time between ALS onset and TTS development was 5.8 ± 3.4 (range, 2.8–10) years. Two patients underwent tracheostomy-invasive ventilation (TIV). Only 1 patient reported chest pain and 3 patients were complicated by acute infections. One patient died acutely after the development of TTS.

### Case Report

#### Case 1

A 71-year-old woman presented with a chief complaint of dysarthria and was subsequently diagnosed with ALS. At age 75, her breathing became difficult, and she underwent TIV. At age 78, she was hospitalized due to fever, tachycardia, hypoxia, and drowsiness. She had pyuria and increased white blood cells and C-reactive protein. An ECG showed ST elevation in V1–V5. Echocardiography demonstrated markedly decreased wall motion in the apex, which was incongruent with the coronary artery supply region. The basal motion was normal. She was diagnosed with a urinary tract infection and TTS. Eleven days after admission, the abnormal wall motion and her symptoms completely disappeared.

#### Case 2

A 57-year-old man presented with right arm weakness and was diagnosed with ALS. At age 60, he underwent TIV due to progressive dyspnea. At age 67, he was admitted to our hospital due to respiratory discomfort. An ECG showed negative T waves in V1-V6, and echocardiography indicated decreased wall motion over the entire circumference of the apical region. Blood testing showed increased white blood cells, transaminase, gamma-glutamyl transpeptidase, and C-reactive protein. Abdominal CT demonstrated wall thickening of the common bile duct, which was consistent with acute cholangitis. The cholangitis was successfully treated with antibiotics. The wall motion abnormality and his symptoms completely disappeared, and he was discharged from our hospital after 27 days.

#### Case 3

**Figure 1:**
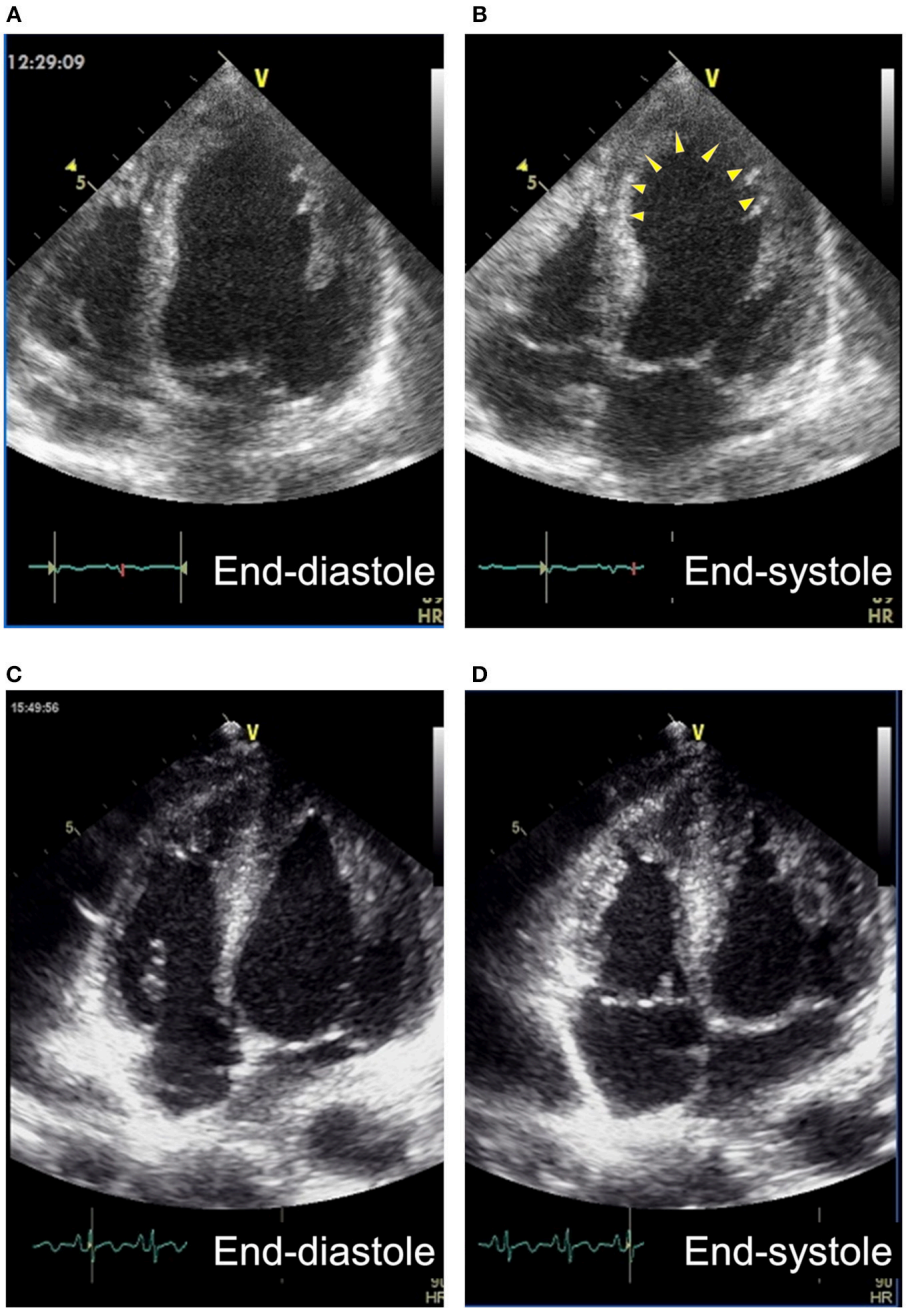
Apical 4-chamber views of transthoracic echocardiography of Case 3. **(A,B)** The broad anterior wall including apex (arrowheads) showed akinesis at the onset. **(C,D)** Myocardium of the region became hypertrophied and showed normal wall motion 2 weeks after the onset.

**Figure 2:**
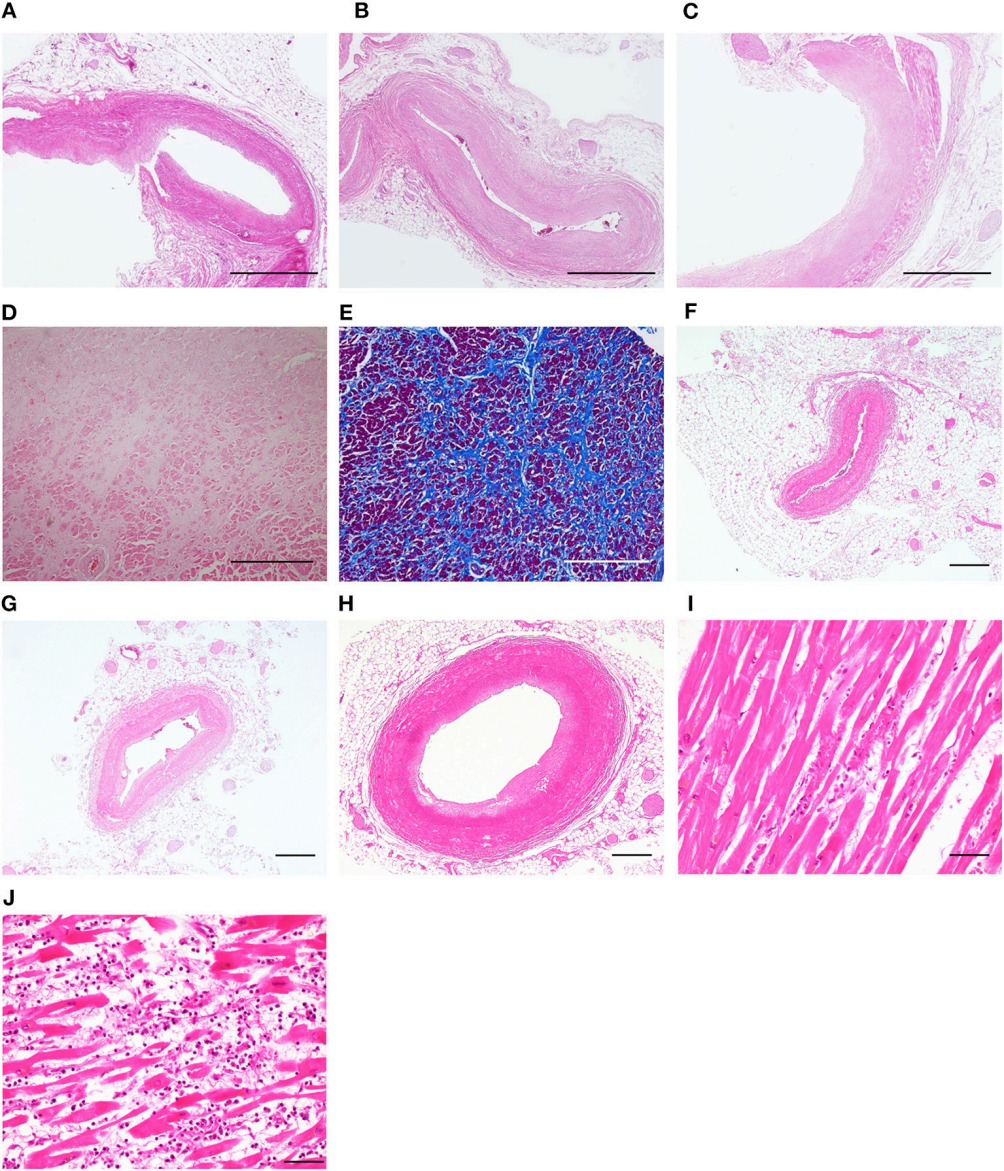
Pathological findings of the heart. Case 3: no occlusion was observed in the left anterior descending coronary artery **(A)**, the left circumflex coronary artery **(B)**, and the right coronary artery **(C)**. Patchy fibrosis was observed in the left anterior wall **(D,E)**. Case 4: no occlusion was observed in the left anterior descending coronary artery **(F)**, the left circumflex coronary artery **(G)**, and the right coronary artery **(H)**. Myocardial necrosis was observed in the anterior wall of the apex **(H)** and the intraventricular septum **(J)**, accompanied by neutrophils and lymphocytes **(A–D,F–J)** hematoxylin and eosin staining. **(E)** Azan staining. Bars = 1 mm for **(A–C)**, 500 μm for **(D–H)** and 50 μm for **(I,J)**.

**Figure 3:**
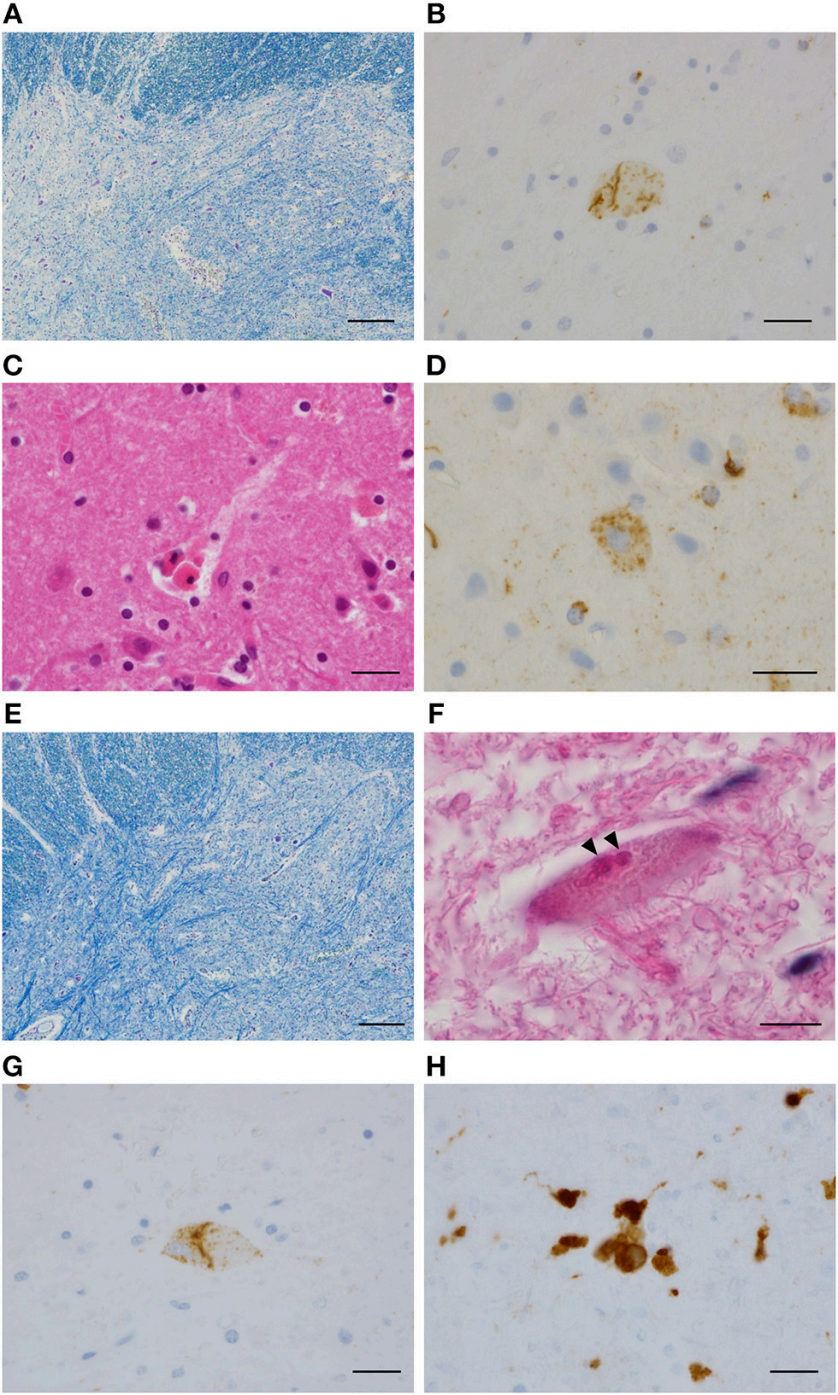
Neuropathological findings of Case 3 and Case 4. Case 3: severe neuronal loss in the cervical anterior horn **(A)**; phosphorylated TDP-43 immunopositive neuronal cytoplasmic inclusions in the cervical anterior horn **(B)**; neuronophagia **(C)**, and phosphorylated TDP-43 immunopositive neuronal and glial cytoplasmic inclusions **(D)** in the primary motor area. Case 4: severe neuronal loss in the lumbar anterior horn **(E)**; Bunina body (**F**, arrowhead) and phosphorylated TDP-43 immunopositive neuronal cytoplasmic inclusions **(G)** in the lumbar anterior horn; neuronophagia **(H)** in the primary motor area. **(A,E)** Klüver-Barrera staining. **(B,D,G)** Phosphorylated TDP-43 immunostaining. **(C,F)** Hematoxylin and eosin staining. **(H)** CD68 immunostaining. Bars = 200 μm for **(A,E)**, 20 μm for **(B–D,G–H)**, and 10 μm for **(F)**.

A 60-year-old man presented with weakness in the left foot. He subsequently developed dysarthria and swallowing difficulties and was diagnosed with ALS. At age 63, he was admitted to our hospital with fever and exacerbation of swallowing difficulties, and he was diagnosed with aspiration pneumonia. Two days after admission, he reported chest pain. An ECG indicated negative T waves in V3–V6. An echocardiogram indicated severe left ventricular dysfunction with akinesia; however, the basal segments were preserved (Figures 1A,B, Supplementary Video 1). The ejection fraction was 20%. Coronary angiography did not indicate any significant stenosis. Although his chest pain subsided with conservative treatment, he underwent tracheostomy due to difficulties in sputum expectoration. The abnormal wall motion was subsequently resolved (Figures 1C,D, Supplementary Video 2). He was transferred to another hospital and died at 65 years of age. Pathological examination of the heart demonstrated no coronary stenosis (Figures 2A–C) and localized patchy fibrosis that occurred toward the endocardium of the left anterior wall (Figures 2D,E). The neuropathological findings were consistent with ALS [Brettschneider stage 4 (16) and Nishihira Type 2 (17)] (Figures 3A–D).

#### Case 4

A 52-year-old woman presented with dysarthria, followed by swallowing difficulties and limb weakness, and she was diagnosed with ALS. At age 55, she developed hypoxia and consciousness disturbance after excreting a large amount of stool induced by an enema. An ECG indicated abnormal Q wave in V3 and ST elevation in V2–V3. Echocardiogram revealed diffuse akinesia in the apex with preserved wall motion in the basal segments. She died of hypoxia on the following day. Pathological examination of the heart demonstrated no apparent occlusion of the coronary artery (Figures 2F–H). Patchy myocardial necrosis was observed in the anterior wall of the apex and the intraventricular septum, accompanied by inflammatory cell infiltration that mainly involved neutrophils and lymphocytes (Figures 2I,J). Intraventricular hemorrhage was also observed. The neuropathological findings were consistent with ALS (Brettschneider stage 4 and Nishihira Type 1) (Figures 3E–H).

### Literature Review

We identified 8 reports that addressed ALS patients complicated with TTS and 1 report each for PD and DLB with TTS, including 16 TTS cases for ALS and 1 case each for PD and DLB (10, 18–26). Table 1 shows a summary of 20 cases of ALS complicated with TTS, including 4 cases from this study. Of these 20 cases, 9 (45%) cases were male and 11 (55%) cases were female. The onset types of ALS were bulbar in 7 (35%) cases, cervical in 7 (35%) cases, lumbar in 2 (10%) cases, dyspnea in 1 (5%) case, and not clearly indicated in 3 (15%) cases. The mean age of TTS onset was 63.3 ± 9.0 (range, 40–75) years, and the mean interval time from ALS onset to TTS development was 4.9 ± 3.0 (1.5–11.2) years. Ten (50%) patients underwent TIV or non-invasive ventilation (NIV). The predisposing or precipitating factors of TTS varied; however, TTS development was associated with surgery or interventional endoscopy in 5 (25%) patients. Four (20%) cases, including 3 of our cases, were complicated by various acute infections. Among 18 patients whose prognoses were documented, 3 (17%, including 2 women) patients died of TTS.

**Table 1:** Clinical profile of ALS patients with TTS.

**Age at TTS onset**	**Sex**	**Onset type of ALS**	**Duration before TTS (mo)**	**Artificial ventilation**	**Potential predisposing/ precipitating factors**	**Cardiac motion abnormality**	**Recovery from TTS**	**References**
60	F	Cervical	43	None	ND	Apical hypokinesia	ND	(10)
71	M	Bulbar	18	NIV	PEG installation	Mid-apical ballooning with basal hypercontractility	Partial	(10)
57	M	Cervical	18	None	ND	Akinesia at whole apex	Full	(10)
59	F	Cervical	25	None	ND	Whole mid to apical wall akinesia	Full	(10)
70	F	Bulbar	63	NIV	^†^	Apical and mid-ventricular akinesia	Partial	(10)
69	F	Bulbar	60	TIV	ND	Apical and mid ventricular akinesia with hyperdynamic basal segments	Full	(10)
59	M	Cervical	69	NIV	^†^	Apical akinesia with ballooning	Full	(10)
70	F	Cervical	134	TIV	^†^	Apico-anterior akinesia	Full	(10)
55	M	Dyspnea	ND	None	Colorectal cancer with liver metastasis, Postoperative period	Focal akinesia of mid inferolateral wall	Full	(10)
74	F	ND	ND	None	First attack: emotional stress	Apical ballooning and basal hyperkinesis	Full	(20)
–	–	–	–	–	Second attack: surgical stress and hypovolemia	Apical ballooning with systolic anterior motion of the mitral valve	Full	–
75	F	Bulbar	ND	None	Surgical gastrostomy and hernia repair	Apico-anterior hypokinesia with hyperdynamic basal segments	Full	(21)
59	F	Bulbar	93	TIV	ND	Apical akinesia with hyperdynamic basal segments	Full	(22)
63	M	Cervical	86	None	Aspiration pneumonia and tracheostomy	Apical hypokinesia with hyperdynamic basal segments	Dead	(23)
40	M	ND	24	NIV	ND	Typical apical ballooning	Full	(24)
71	F	Lumbar	71	None	Difficulty with discharging sputum	Apical hypokinesia with hyperdynamic basal segments	Dead	(25)
61	M	ND	ND	NIV	ND	Apical ballooning	ND	(26)
78	F	Bulbar	85	TIV	Urinary tract infection	Apical akinesia	Full	Case 1
67	M	Cervical	120	TIV	Cholangitis	Akinesia at whole apex	Full	Case 2
63	M	Lumbar	34	None	Aspiration pneumonia	Apico-anterior hypokinesia	Full	Case 3
55	F	Bulbar	38	None	Evacuation	Diffuse akinesia in the apex	Dead	Case 4

† *Treated with benzodiazepines, anxiolytics, or tricyclic antidepressants. Ref, reference; ND, not described; NIV, noninvasive ventilation; TIV, tracheostomy-invasive ventilation; PEG, percutaneous endoscopic gastrostomy*.

A reported case with PD developed TTS after initiating entacapone, a cardiac catechol-O-methyltransferase (c-COMT) inhibitor (18). The authors suggested a potential relationship between TTS and entacapone intake as the inhibition of c-COMT activity with entacapone increases noradrenaline, adrenaline, and dopamine bioavailability. The case with DLB, who showed decreased cardiac (123I) metaiodobenzyl guanidine (MIBG) uptake, developed TTS without chest pain (19). With the exception of depression, the apparent precipitating factors were not described in the report.

## Discussion

### TTS in ALS

Regarding the incidence of TTS in ALS, Choi et al. investigated 64 of 624 ALS patients who exhibited chest symptoms and identified 9 (1.4% of overall patients and 14.1% of patients with positive chest symptoms) patients with TTS (10). The incidence rate was close to that of our current study (1.6%). Here, we further confirmed that ALS is a substantial risk for developing TTS, considering only 0.02% of all hospitalizations in the United States (*n* = 33,506,402) were diagnosed with TTS (27).

TTS is associated with catecholamine excess (3), and exposure to catecholamines and beta-receptor agonists precipitates all features of TTS in humans (4). Biopsy samples from TTS patients demonstrated morphological changes compatible with catecholamine-induced myocardial damage (5–7), and the apical myocardium of the left ventricle has a high density of β-adrenoceptors, being particularly sensitive to catecholamines (8). Moreover, evidence has demonstrated impaired autonomic nervous systems in ALS patients. Patients in the early stage of ALS may have a higher resting heart rate and blood pressure and an increased plasma noradrenaline level, which indicate an increased sympathetic tone (28, 29). In the advanced stage requiring artificial ventilations, hypertensive crisis may occur (30). These findings indicate that the high incidence of TTS in ALS is due to sympathetic overactivity.

A recent review indicated that cardiac sympathetic overactivity and local noradrenaline spillover may be fundamental in the etiology of TTS (9). Interestingly, a model of sudden brain death demonstrated massive increases in myocardial interstitial noradrenaline levels; however, the serum catecholamine remained relatively unchanged (31). Similarly, sympathectomy, but not adrenalectomy, prevented a left ventricular motion abnormality in baboons after subarachnoid hemorrhage (32). Importantly, ALS patients show chronic cardiac sympathetic hyperactivity as evidenced by an increased washout ratio of cardiac MIBG scintigraphy (11). These observations may jointly support that local noradrenaline release associated with cardiac sympathetic overactivity is more crucial than the systemic catecholamine increase in TTS development.

Of the 20 patients with ALS complicated with TTS in the current and previous reports, one half of the patients underwent TIV or NIV. The use of artificial ventilation is likely to become physical and psychological burdens in addition to the disease burden. However, we could not determine whether there is an additional risk of TTS from this result. The mortality rate was 17%, which was substantially higher than that obtained from a meta-analysis (4.5%) (33), which likely reflects the diminished respiratory reserve in ALS. Although a retrospective study of a large administrative database demonstrated that male patients with TTS had a higher mortality rate than females (34), among 3 patients who died, 2 were female. Given that none of these 3 patients underwent TIV or NIV, which is occasionally required to treat acute complications of TTS, we speculate that the decision whether to use artificial ventilation may be more associated with mortality than sex in ALS patients.

In contrast to the significant female preponderance in the general population of TTS (27, 35), the number of female ALS patients only slightly exceeded that of the male ALS patients. This finding is partially explained by the gender difference of ALS (the ratio of affected males to affected females is 1.6:1) (36); however, it may be influenced by unknown factors. The time period between the ALS onset and development of TTS was documented in 16 patients. Among these patients, 11 patients developed TTS 3 or more years after ALS onset, and no patients developed TTS within 12 months. This finding suggests that the risk may increase over time and may relate to the severe autonomic symptoms observed in an advanced stage (30). Although brain autonomic dysfunction in ALS potentially contribute to TTS (29), the number of bulbar-onset patients did not outweigh that of the limb-onset patients.

Most cases had precipitating factors, and TTS development was associated with gastrostomy, tracheostomy, or infections in approximately half of the patients. Interestingly, acute infections apparently precipitated TTS in 3 of 4 our cases. Although acute infectious diseases can trigger TTS in some patients (37), an infection *per se* is unlikely to cause TTS considering its prevalence. Therefore, the etiology of TTS in these 3 cases may be best explained by the two-hit theory; acute infection precipitated TTS under the presence of chronic cardiac sympathetic overactivity in ALS. This hypothesis seems to fit clinical presentations in the majority of ALS patients with TTS, thus illustrating the etiological differences from acute-onset or paroxysmal CNS disorders, in which the disease *per se* causes TTS.

### TTS in Synucleinopathies

Synucleinopathies (PD, DLB, and MSA) are common neurodegenerative diseases characterized by abnormal accumulation of the aggregates of α-synuclein in neurons, nerve fibers, or glial cells. Although they are substantially more prevalent than ALS, our literature review identified only one case each for PD and DLB accompanied by TTS (18, 19). More interestingly, there was no report on TTS that occurred under the presence of MSA, which typically progresses more rapidly than PD or DLB and is often considered the most devastating type of synucleinopathy. In contrast to ALS, autonomic impairments in synucleinopathies present with constipation, urinary impairments, and orthostatic hypotension, which typically result from a reduced sympathetic tone. In PD and DLB, functional impairments of cardiac autonomic nerves can be visualized by MIBG scintigraphy; most patients with PD and DLB show significantly decreased cardiac uptake of MIBG, which reflects postsynaptic cardiac noradrenergic denervation and is used as an established diagnostic marker (38). Moreover, ~30% of patients with MSA exhibit a low MIBG uptake (39).

Here, we found that no patients with synucleinopathies developed TTS in our cohort, which is in line with the limited number of previous reports on synucleinopathies accompanying TTS. The considerable difference in the TTS incidence between synucleinopathies and ALS may solely arise from the sympathetic overactivity in ALS. Moreover, cardiac sympathetic denervation in synucleinopathies might widen the incidence gap. To support this idea, pharmacological and surgical sympathectomies have successfully prevented the induced myocardial changes that resemble those of TTS in animal models (32, 40). Future clinical studies that focus on the potential effects of pre-existing cardiac sympathetic denervation may clarify whether or to what extent it is protective against the development of TTS in human diseases.

### Pathological Examination

Two patients underwent autopsies (Cases 3 and 4) and coronary artery occlusion was not observed in these cases, which further supports the diagnosis of TTS. According to a histopathological study on 11 TTS cases (6), TTS is characterized by: (a) an immediate infiltration of inflammatory cells including neutrophils, followed by (b) the removal of cardiomyocytes, and then (c) patchy fibrosis. These pathological features including time-course changes are consistent with catecholamine-induced cardiomyopathy (7). We observed an infiltration of neutrophils in Case 4, who died the following day after TTS, and only patchy focal fibrosis in Case 3, who underwent an autopsy 2 years after TTS onset. In Case 4, patchy necrosis and intraventricular hemorrhage were also observed, which resemble the pathological findings in severe TTS cases who died acutely after the development of TTS (41, 42).

### Limitations

As this cohort was not originally created to survey sympathetic function or mental status in neurodegenerative diseases, we did not perform cardiac MIBG scintigraphy in ALS patients, or assess the blood catecholamine, anxiety or depression levels. Moreover, we might have underestimated the incidence of TTS in ALS and synucleinopathies. ALS patients, particularly patients who are on artificial ventilations, often have difficulties in reporting subjective symptoms. Similarly, cardiac sympathetic denervation in synucleinopathies might mask chest symptoms; sympathetic autonomic neuropathy has been reported to be associated with pure silent ischemia (43), and the DLB patient with TTS did not present chest symptoms (19). Improved recognition of the TTS risk in ALS as well as possible silent TTS in synucleinopathies may elucidate a more accurate incidence rate in future studies.

## Conclusion

This cohort study confirmed that ALS is a considerable predisposing factor of TTS and demonstrated that synucleinopathies rarely develop TTS. Our findings also provide unique opportunities to disentangle the complex etiology of TTS; local noradrenaline release associated with cardiac sympathetic overactivity seems to be the most crucial predisposing factor in ALS, and TTS may not occur until the “second-hit,” such as when an infection or surgical intervention occurs. Although the potential effect of pre-existing cardiac sympathetic denervation has rarely been discussed in the clinical context, it might affect the development and disease severity of TTS. Further detailed research on the TTS incidence in different types of diseases will provide new insights into the etiology of TTS.

## Author Contributions

YI designed the project, analyzed the data, drafted the manuscript, and oversaw the project. RM, KF, YY, HY, TM, YU, AT, HN, SH, MO, AM, and YN interpreted the data and drafted the manuscript. NT performed the sonography. TM, SW, HO, HU, and SM performed the pathological analysis and drafted the manuscript. MS and RK supervised this study.

### Conflict of Interest Statement

The authors declare that the research was conducted in the absence of any commercial or financial relationships that could be construed as a potential conflict of interest.

## Supplementary Material

Supplementary Video 1 Apical 4-chamber views of transthoracic echocardiography at the onset in Case 3.

Supplementary Video 2 Apical 4-chamber views of transthoracic echocardiography 2 weeks after the onset in Case 3.

## References

[1] Akashi YJ Nef HM Lyon AR. Epidemiology and pathophysiology of Takotsubo syndrome. Nat Rev Cardiol. (2015) 12:387–97. 10.1038/nrcardio.2015.39 25855605

[2] Ghadri JR Wittstein IS Prasad A Sharkey S Dote K Akashi YJ. International expert consensus document on takotsubo syndrome (Part I): clinical characteristics, diagnostic criteria, and pathophysiology. Eur Heart J. (2018) 39:2032–46. 10.1093/eurheartj/ehy076 29850871 PMC5991216

[3] Finsterer J Wahbi K. CNS disease triggering Takotsubo stress cardiomyopathy. Int J Cardiol. (2014) 177:322–9. 10.1016/j.ijcard.2014.08.101 25213573

[4] Abraham J Mudd JO Kapur NK Klein K Champion HC Wittstein IS. Stress cardiomyopathy after intravenous administration of catecholamines and beta-receptor agonists. J Am Coll Cardiol. (2009) 53:1320–5. 10.1016/j.jacc.2009.02.020 19358948

[5] Nef HM Mollmann H Kostin S Troidl C Voss S Weber M. Tako-Tsubo cardiomyopathy: intraindividual structural analysis in the acute phase and after functional recovery. Eur Heart J. (2007) 28:2456–64. 10.1093/eurheartj/ehl570 17395683

[6] Kawai S Ampulla-shaped ventricular dysfunction or ampulla cardiomyopathy? Kokyu Junkan (2000) 48:1237–48.

[7] Haft JI. Cardiovascular injury induced by sympathetic catecholamines. Prog Cardiovasc Dis. (1974) 17:73–86. 10.1016/0033-0620(74)90039-5 4599470

[8] Paur H Wright PT Sikkel MB Tranter MH Mansfield C O'Gara P. High levels of circulating epinephrine trigger apical cardiodepression in a beta2-adrenergic receptor/Gi-dependent manner: a new model of Takotsubo cardiomyopathy. Circulation (2012) 126:697–706. 10.1161/CIRCULATIONAHA.112.111591 22732314 PMC4890655

[9] Y-Hassan S. Acute cardiac sympathetic disruption in the pathogenesis of the takotsubo syndrome: a systematic review of the literature to date. Cardiovasc Revasc Med. (2014) 15:35–42. 10.1016/j.carrev.2013.09.008 24140050

[10] Choi SJ Hong YH Shin JY Yoon BN Sohn SY Park CS. Takotsubo cardiomyopathy in amyotrophic lateral sclerosis. J Neurol Sci. (2017) 375:289–93. 10.1016/j.jns.2017.02.012 28320151

[11] Tanaka Y Yamada M Koumura A Sakurai T Hayashi Y Kimura A. Cardiac sympathetic function in the patients with amyotrophic lateral sclerosis: analysis using cardiac [123I] MIBG scintigraphy. J Neurol. (2013) 260:2380–6. 10.1007/s00415-013-7005-0 23784610

[12] Brooks BR Miller RG Swash M Munsat TL World federation of neurology research group on motor neuron diseases. El Escorial revisited: revised criteria for the diagnosis of amyotrophic lateral sclerosis. Amyotroph Lateral Scler Other Motor Neuron Disord. (2000) 1:293–9. 10.1080/146608200300079536 11464847

[13] Hughes AJ Daniel SE Kilford L Lees AJ. Accuracy of clinical diagnosis of idiopathic Parkinson's disease: a clinico-pathological study of 100 cases. J Neurol Neurosurg Psychiatry (1992) 55:181–4. 10.1136/jnnp.55.3.181 1564476 PMC1014720

[14] McKeith IG Dickson DW Lowe J Emre M O'Brien JT Feldman H. Diagnosis and management of dementia with Lewy bodies: third report of the DLB Consortium. Neurology (2005) 65:1863–72. 10.1212/01.wnl.0000187889.17253.b1 16237129

[15] Gilman S Wenning GK Low PA Brooks DJ Mathias CJ Trojanowski JQ. Second consensus statement on the diagnosis of multiple system atrophy. Neurology (2008) 71:670–6. 10.1212/01.wnl.0000324625.00404.15 18725592 PMC2676993

[16] Brettschneider J Del Tredici K Toledo JB Robinson JL Irwin DJ Grossman M. Stages of pTDP-43 pathology in amyotrophic lateral sclerosis. Ann Neurol. (2013) 74:20–38. 10.1002/ana.23937 23686809 PMC3785076

[17] Nishihira Y Tan CF Onodera O Toyoshima Y Yamada M Morita T. Sporadic amyotrophic lateral sclerosis: two pathological patterns shown by analysis of distribution of TDP-43-immunoreactive neuronal and glial cytoplasmic inclusions. Acta Neuropathol. (2008) 116:169–82. 10.1007/s00401-008-0385-z 18481073

[18] Baldacci F Vergallo A Del Dotto P Ulivi M Palombo C Casolo G. Occurrence of Takotsubo syndrome in a patient with Parkinson's disease after entacapone add-on. Parkinsonism Relat Disord. (2014) 20:1313–4. 10.1016/j.parkreldis.2014.09.009 25258328

[19] Noguchi M Yamaga K. A patient with possible dementia with Lewy bodies (DLB) who presented with Takotsubo cardiomyopathy. Psychiatry Clin Neurosci. (2010) 64:213. 10.1111/j.1440-1819.2010.02068.x 20447019

[20] Santoro F Ieva R Ferraretti A Carapelle E De Gennaro L Specchio LM. Early recurrence of Tako-Tsubo cardiomyopathy in an elderly woman with amyotrophic lateral sclerosis: different triggers inducing different apical ballooning patterns. J Cardiovasc Med. (2016) 17(Suppl. 2):e266–8. 10.2459/JCM.0b013e328364dcbc 28079765

[21] Takayama N Iwase Y Ohtsu S Sakio H. [“Takotsubo” cardiomyopathy developed in the postoperative period in a patient with amyotrophic lateral sclerosis]. Masui (2004) 53:403–6. 15160667

[22] Mitani M Funakawa I Jinnai K. [Transient left ventricular apical ballooning, “Takotsubo” cardiomyopathy, in an amyotrophic lateral sclerosis patient on long-term respiratory support]. Rinsho Shinkeigaku (2005) 45:740–3. 16318369

[23] Matsuyama Y Sasagasako N Koike A Matsuura M Koga T Kawajiri M. [An autopsy case of amyotrophic lateral sclerosis with ampulla cardiomyopathy]. Rinsho Shinkeigaku (2008) 48:249–54. 10.5692/clinicalneurol.48.249 18453156

[24] Massari FM Tonella T Tarsia P Kirani S Blasi F Magrini F. [Tako-tsubo syndrome in a young man with amyotrophic lateral sclerosis. a case report]. G Ital Cardiol. (2011) 12:388–91. 10.1714/643.7506 21593960

[25] Suzuki Y Oishi M Kanno A Ogawa K Fujisawa M Kamei S. Amyotrophic lateral sclerosis accompanying elevated catecholamines occurring as a complication of takotsubo cardiomyopathy. Geriatr Gerontol Int. (2013) 13:240–1. 10.1111/j.1447-0594.2012.00943.x 23286571

[26] Peters S. Tako tsubo cardiomyopathy in respiratory stress syndrome in amyotrophic lateral sclerosis. Int J Cardiol. (2014) 177:187. 10.1016/j.ijcard.2014.09.135 25499374

[27] Deshmukh A Kumar G Pant S Rihal C Murugiah K Mehta JL. Prevalence of Takotsubo cardiomyopathy in the United States. Am Heart J. (2012) 164:66–71e1. 10.1016/j.ahj.2012.03.020 22795284

[28] Baltadzhieva R Gurevich T Korczyn AD. Autonomic impairment in amyotrophic lateral sclerosis. Curr Opin Neurol. (2005) 18:487–93. 10.1097/01.wco.0000183114.76056.0e 16155429

[29] Shimizu T Sympathetic hyperactivity and sympathovagal impalance in amyotrophic lateral sclerosis. Eur Neurol Rev. (2013) 8:46–50. 10.17925/ENR.2013.08.01.46

[30] Shimizu T Hayashi H Kato S Hayashi M Tanabe H Oda M. Circulatory collapse and sudden death in respirator-dependent amyotrophic lateral sclerosis. J Neurol Sci. (1994) 124:45–55. 10.1016/0022-510X(94)90009-4 7931421

[31] Mertes PM Carteaux JP Jaboin Y Pinelli G el Abassi K Dopff C. Estimation of myocardial interstitial norepinephrine release after brain death using cardiac microdialysis. Transplantation (1994) 57:371–7. 10.1097/00007890-199402150-00010 8108872

[32] Novitzky D Wicomb WN Cooper DK Rose AG and Reichart B. Prevention of myocardial injury during brain death by total cardiac sympathectomy in the Chacma baboon. Ann Thorac Surg. (1986) 41:520–4. 10.1016/S0003-4975(10)63032-9 3707246

[33] Singh K Carson K Shah R Sawhney G Singh B Parsaik A. Meta-analysis of clinical correlates of acute mortality in takotsubo cardiomyopathy. Am J Cardiol. (2014) 113:1420–8. 10.1016/j.amjcard.2014.01.419 24685327

[34] Brinjikji W El-Sayed AM Salka S. In-hospital mortality among patients with takotsubo cardiomyopathy: a study of the National Inpatient Sample 2008 to 2009. Am Heart J. (2012) 164:215–21. 10.1016/j.ahj.2012.04.010 22877807

[35] Murakami T Yoshikawa T Maekawa Y Ueda T Isogai T Konishi Y. Characterization of predictors of in-hospital cardiac complications of takotsubo cardiomyopathy: multi-center registry from Tokyo CCU Network. J Cardiol. (2014) 63:269–73. 10.1016/j.jjcc.2013.09.003 24139869

[36] Mitchell JD Borasio GD. Amyotrophic lateral sclerosis. Lancet (2007) 369:2031–41. 10.1016/S0140-6736(07)60944-1 17574095

[37] De Giorgi A Fabbian F Pala M Parisi C Misurati E Molino C. Takotsubo cardiomyopathy and acute infectious diseases: a mini-review of case reports. Angiology (2015) 66:257–61. 10.1177/0003319714523673 24576981

[38] Orimo S Ozawa E Nakade S Sugimoto T Mizusawa H. (123)I-metaiodobenzylguanidine myocardial scintigraphy in Parkinson's disease. J Neurol Neurosurg Psychiatry (1999) 67:189–94. 10.1136/jnnp.67.2.189 10406987 PMC1736461

[39] Nagayama H Ueda M Yamazaki M Nishiyama Y Hamamoto M Katayama Y. Abnormal cardiac [(123)I]-meta-iodobenzylguanidine uptake in multiple system atrophy. Mov Disord. (2010) 25:1744–7. 10.1002/mds.23338 20645402

[40] McNair JL Clower BR Sanford RA. The effect of reserpine pretreatment on myocardial damage associated with simulated intracranial hemorrhage in mice. Eur J Pharmacol. (1970) 9:1–6. 10.1016/0014-2999(70)90312-2 5434290

[41] Kinbara T Hayano T Otani N Furutani Y Murakami T Yano M An autopsy case of tako-tsubo cardiomyopathy presenting ventricular tachycardia after pacemaker implantation. J Cardiol Cases (2013) 8:134–7. 10.1016/j.jccase.2013.06.007 30546765 PMC6281519

[42] Sacha J Maselko J Wester A Szudrowicz Z Pluta W. Left ventricular apical rupture caused by takotsubo cardiomyopathy–comprehensive pathological heart investigation. Circ J. (2007) 71:982–5. 10.1253/circj.71.982 17527000

[43] Shakespeare CF Katritsis D Crowther A Cooper IC Coltart JD Webb-Peploe MW. Differences in autonomic nerve function in patients with silent and symptomatic myocardial ischaemia. Br Heart J. (1994) 71:22–9. 10.1136/hrt.71.1.22 8297687 PMC483603

